# Proteolytic Activity of Prostate-Specific Antigen (PSA) towards Protein Substrates and Effect of Peptides Stimulating PSA Activity

**DOI:** 10.1371/journal.pone.0107819

**Published:** 2014-09-19

**Authors:** Johanna M. Mattsson, Suvi Ravela, Can Hekim, Magnus Jonsson, Johan Malm, Ale Närvänen, Ulf-Håkan Stenman, Hannu Koistinen

**Affiliations:** 1 Department of Clinical Chemistry, Biomedicum Helsinki, University of Helsinki and Helsinki University Central Hospital, Helsinki, Finland; 2 Department of Laboratory Medicine, Section for Clinical Chemistry, Lund University and Laboratory Medicine Skåne, Lund, Sweden; 3 School of Pharmacy, University of Eastern Finland, Kuopio, Finland; Southern Illinois University School of Medicine, United States of America

## Abstract

Prostate-specific antigen (PSA or kallikrein-related peptidase-3, KLK3) exerts chymotrypsin-like proteolytic activity. The main biological function of PSA is the liquefaction of the clot formed after ejaculation by cleavage of semenogelins I and II in seminal fluid. PSA also cleaves several other substrates, which may explain its putative functions in prostate cancer and its antiangiogenic activity. We compared the proteolytic efficiency of PSA towards several protein and peptide substrates and studied the effect of peptides stimulating the activity of PSA with these substrates. An endothelial cell tube formation model was used to analyze the effect of PSA-degraded protein fragments on angiogenesis. We showed that PSA degrades semenogelins I and II much more efficiently than other previously identified protein substrates, e.g., fibronectin, galectin-3 and IGFBP-3. We identified nidogen-1 as a new substrate for PSA. Peptides B2 and C4 that stimulate the activity of PSA towards small peptide substrates also enhanced the proteolytic activity of PSA towards protein substrates. Nidogen-1, galectin-3 or their fragments produced by PSA did not have any effect on endothelial cell tube formation. Although PSA cleaves several other protein substrates, in addition to semenogelins, the physiological importance of this activity remains speculative. The PSA levels in prostate are very high, but several other highly active proteases, such as hK2 and trypsin, are also expressed in the prostate and may cleave protein substrates that are weakly cleaved by PSA.

## Introduction

Proteases have a significant influence on tumor growth, invasion and formation of metastases [1]. Increased proteolytic activity in the tumor microenvironment has been associated with cancer progression, as degradation of extracellular matrix components is needed for cancer cell migration and invasion [2]. Proteases may also exert several other functions relevant in cancer, including activation of protease activated receptors (PARs) and regulation of the activity of other signaling molecules, such as kinases and growth factors [3], [4]. However, the role of proteases in cancer is not straightforward, as individual proteases interact with each other in complex proteolytic networks, and some proteases have been found to have tumor-suppressive functions [1], [2], [5], thus, besides facilitating cell invasion, matrix metalloproteinases may also inhibit cancer cell growth, induce apoptosis and produce antiangiogenic fragments from plasminogen and collagen type IV [6], [7].

The prostate produces several proteases, the expression of which varies during prostate cancer development [8], [9]. The most abundant protease expressed in the prostate and secreted into the seminal fluid is prostate-specific antigen (PSA or kallikrein-related peptidase-3, KLK3), a well-known biomarker of prostate cancer [10]–[12]. In spite of its clinical importance, the functional role of PSA in prostate cancer is not well understood [9], [13]. PSA is a 28 kDa serine protease with chymotrypsin-like enzymatic activity [14], [15]. The known physiological function of PSA is degradation of the gel-forming proteins semenogelins I and II in semen after ejaculation [16]. This leads to liquefaction of the seminal clot and the release of motile sperm, thus enabling the spermatozoa to travel through the female reproductive tract [17], [18]. Also, several other functions have been suggested for PSA, most of which depend on its proteolytic activity [8], [9]. These proposed functions have mainly been studied using *in vitro* methods and it is not known whether they are relevant for prostate cancer development or normal physiology.

Several studies have demonstrated that PSA exerts antiangiogenic activity *in vitro* and *in vivo* [19]–[22]. By using an endothelial cell tube formation model, we have shown that the enzymatic activity of PSA is essential for this activity [23]. Previously, PSA has been shown to cleave components of the extracellular matrix (ECM), e.g., laminin and fibronectin, as well as unidentified proteins in the basement membrane preparation Matrigel [24]–[26]. Proteolytic products of some ECM proteins are known to inhibit angiogenesis [27], but degradation of ECM is also crucial for cancer cell invasion.

Previously, we have used phage display to identify peptides that bind to PSA and stimulate its proteolytic activity towards a small chromogenic substrate [28]. These peptides also enhance the antiangiogenic activity of PSA in the tube formation model [29] and have been suggested to be useful lead molecules for the development of new prostate cancer treatments [30].

In this study we compared the proteolytic activity of PSA, with and without the PSA-activity stimulating peptides, towards two small peptide substrates and several putative protein substrates. By studying protein fragments cleaved by PSA in Matrigel we showed that nidogen-1 (also known as entactin), a linker protein in the basement membrane [31], is a novel PSA substrate. As nidogen-1 and galectin-3 have been associated with angiogenesis, we studied whether they or their proteolytic fragments would affect angiogenesis in an endothelial cell tube formation model. Comparison of the cleavage of different protein substrates by PSA showed that semenogelins I and II were degraded much more efficiently than other protein substrates. Thus the physiological relevance of the proteolytic activity of PSA towards protein substrates other than semenogelins remains unclear.

## Materials and Methods

### Proteins and peptides

The major form of active PSA (isoform PSA-B) was purified from pooled human seminal fluid by immunoaffinity chromatography [32] followed by anion exchange chromatography [33]. Semenogelins I and II were isolated from human seminal fluid as previously described [34], but instead of alkylating the proteins, the reducing agent 30 mM dithiothreitol (Sigma-Aldrich, St. Louis, MO) was included in all buffers during purification. Semenogelins were stored in the purification buffer containing 2 M urea and 5 mM EDTA at −70°C. Prior to cleavage by PSA, the buffer was exchanged to 50 mM Tris, pH 7.7, containing 0.15 M NaCl (TBS), with Amicon Ultra centrifugal filter units (MW cut-off 30 kDa) (Millipore, Cork, Ireland) at 4°C, and protein concentration was determined by measuring absorbance at 280 nm.

Fibronectin from human plasma (BD Biosciences, Bedford, MA), recombinant human insulin-like growth factor-binding protein-3 (IGFBP-3) (PeproTech, London, UK), recombinant human nidogen-1 and galectin-3 (R&D Systems, Minneapolis, MN), recombinant human matrix metalloproteinase-3 (MMP-3) catalytic domain (Calbiochem, Darmstadt, Germany), bovine α-chymotrypsin (Millipore, Freehold, NJ), plasminogen (Calbiochem and Hyphen BioMed, Neuville-sur-Oise, France) and lys-plasminogen from human plasma (American Diagnostica, Stamford, CT) were commercially obtained. Protein LoBind tubes (Eppendorf AG, Hamburg, Germany) were used in all protein degradation experiments.

Peptides that bind to PSA and stimulate its activity have previously been identified by phage display [28]. The 13-amino acid double cyclic peptide C4 (CVAYCIEHHCWTC, with disulfide bridges between cysteines 1–13 and 5–10), the 12- amino acid cyclic peptide B2 (CVFAHNYDYLVC, with a disulfide bridge between cysteines 1–12) with and without C-terminal amidation (B2-NH_2_ and B2, respectively) and an inactive derivative of peptide B2-NH_2_ (B2-NH_2_-control, CVFAHNADALVC, with a disulfide bridge between cysteines 1–12) were purchased from AnaSpec (San Jose, CA), NeoMPS (Strasbourg, France), Biomatik (Cambridge, Ontario, Canada) or GenScript (Piscataway, NJ) as custom peptides. The purity of the peptides as determined by HPLC was over 95% in all batches. We have not observed any significant variation in the capacity of the peptides to stimulate the PSA activity between different peptide batches and suppliers.

### Enzyme kinetics of PSA

The proteolytic activity of PSA towards small peptide substrates was compared with two different substrates [35]. Both assays were performed in 96-well microplates (PerkinElmer, Turku, Finland) with duplicates in two separate experiments (n = 2) in TBS buffer containing 1 g/l bovine serum albumin (0.1% BSA-TBS). The chromogenic chymotrypsin substrate S-2586 (MeO-Suc-Arg-Pro-Tyr-pNA·HCl) (Chromogenix Instrumentation Laboratory, Milan, Italy) at 0.125–5 mM concentration or 0.125–2 mM fluorogenic PSA substrate 4-morpholinecarbonyl-HSSKLQ-AMC (7-amido-4-methylcoumarin) (custom peptide from JPT Peptide Technologies GmbH, Berlin, Germany) [36] were added to the wells together with 175 nM (5 µg/ml) PSA. The absorbance was measured at 405 nm immediately and at 5 min intervals for 30 min and fluorescence at 355/460 nm (excitation/emission) after 10 min, 20 min, 40 min, 1 h, 2 h and 4 h incubation at room temperature with a Victor 1420 Multilabel plate reader (PerkinElmer). In the fluorogenic assays all wells contained 5% dimethyl sulfoxide (DMSO).

The maximum velocity of the reaction (V_max_) and the Michaelis constant (K_m_), i.e., substrate concentration at which the reaction rate is half of the maximum, were evaluated for PSA with both peptide substrates using Sigma Plot 11 with the Enzyme Kinetics Module 1.3 (SYSTAT Software, Inc., San Jose, CA) and the catalytic constant (k_cat_), i.e., the number of substrate molecules converted to product per time unit, was calculated from the equation k_cat_ = V_max/_[PSA]. The absorbance was converted to molar concentration with the Lambert-Beer equation (A = εcl), using the molar absorptivity of pNA (ε_pNA_ = 8800 M^−1^cm^−1^). Fluorescence (counts per second, cps) was transformed to molar concentrations with SigmaPlot by means of a standard curve for 7-amido-4-methylcoumarin (AMC, Sigma-Aldrich).

The effect of the PSA-stimulating peptides on the activity of PSA was characterized with chromogenic and fluorogenic substrates in two (n = 2) and three (n = 3) separate experiments, respectively. PSA (350 nM) was first preincubated with peptides (0.35–350 µM) for 30 min at room temperature. Then 0.2 mM chromogenic or 0.3 mM fluorogenic substrate was added and absorbance or fluorescence was measured as described above. Fluorogenic assays with the peptides were performed in black 384-well microplates (Corning Inc., Corning, NY) and all wells contained 0.75% DMSO. The velocity of the reaction (V) was calculated from the change of absorbance/min or fluorescence/min at the linear phase of the reaction and converted to molar concentration as described above. V_max (peptide + PSA)_, i.e., the maximal velocity of the reaction with increasing peptide concentrations and given PSA and substrate concentrations, was determined using SigmaPlot, fitting the data to f = y0+a*(1−exp(−b*x)) using non-linear regression. The maximal stimulatory capacity of the peptide, V_max (peptide+PSA)/_V_(PSA)_, was calculated. The peptide concentrations at which the stimulation of PSA was half of the maximum ([peptide]_½Vmax_) and at which PSA activity was increased two-fold when compared to PSA alone ([peptide]_2V_) were also determined.

### Surface plasmon resonance

The binding kinetics and affinity of the peptides stimulating the activity of PSA were studied by surface plasmon resonance (SPR) on a Biacore T100 instrument using Biacore T100 Control Software 2.0.3 (both from GE Healthcare, Uppsala, Sweden). PSA (20 µg/ml, 0.7 µM) and α-chymotrypsin (20 µg/ml, 0.8 µM) were covalently immobilized on the Series S Sensor Chip CM5 (GE Healthcare) by using amine coupling in 10 mM sodium acetate buffer, pH 5.0. The immobilization levels varied in different experiments between 2000–3900 resonance units (RU) for PSA and 1400–2500 RU for chymotrypsin. Chymotrypsin was used as a reference for PSA, i.e., the binding of peptides to the chymotrypsin surface was subtracted as a non-specific interaction from the binding of peptides to the PSA surface in all measurements.

Binding to PSA was studied at various peptide concentrations (1.2–600 µM) in phosphate buffered saline (PBS), pH 7.4 (Lonza, Verviers, Belgium), containing 6.7 mM PO_4_ and 0.15 M NaCl, with a flow rate of 40 µl/min at 25°C. Contact time of the peptides with PSA was 60 s and dissociation time 180 s. In all other steps except the peptide binding the running buffer was supplemented with 0.005% surfactant P20 (GE Healthcare). The chip was washed after each measurement cycle with the running buffer, which removed all peptides. The binding data were analyzed using Biacore T100 Evaluation software 2.0.3. (GE Healthcare) with a Steady State Affinity model. The data from 3 to 4 independent experiments each with two replicates (n = 3 for B2 and n = 4 for B2-NH_2_ and C4) were analyzed by Mann-Whitney U test and *p*-values<0.05 were considered significant.

### Characterization of Matrigel degradation and mass spectrometry

Matrigel basement membrane preparation (BD Biosciences) with a protein concentration of 9.7 mg/ml was diluted 1∶20 in TBS. Matrigel proteins (250 µg/ml) were incubated with PSA (200 µg/ml, 7.1 µM) in a 50 µl volume at 37°C for 48 h. A sample containing 5 µg Matrigel proteins and 4 µg PSA was analyzed by SDS-polyacrylamide gel electrophoresis (SDS-PAGE) followed by silver staining and Western blotting (see below).

For mass spectrometry, selected protein fragments were cut from the silver-stained gel, reduced, alkylated with 55 mM iodoacetamide and digested with modified trypsin (V511A, Promega, Madison, WI) [37]. Peptide fragments were purified using a ZipTip (ZipTip_C18_, Millipore, Billerica, MA), which was wetted and equilibrated according to instructions. After sample application, the tip was washed with 0.1% TFA and peptides eluted with 3 µl of 40% ACN in 0.1% TFA and diluted with 21 µl of 0.1% formic acid (FA). The extracted peptide sample (10 µl) was separated on a RP-HPLC (CapLC, Waters, Manchester, UK) with a C18 trap column (Atlantis dC18, NanoEase Trap Column, 5 µm, Waters) and a 0.075×150 mm C18 analytical column (Atlantis dC18, 100 Å, 3 µm, Waters) and eluted with a linear gradient of ACN (from 5 to 50% in 30 min) in 0.1% FA at a flow rate of 0.3 µl/min. MS analysis was performed online with a Q-TOF Micro (Waters) with an ESI source. Fragmentation spectra of the peptides were acquired by colliding doubly or triply charged precursor ions with argon collision gas at accelerating voltages of 20–70 V. The MS was calibrated using 2 pmol/µl glufibrinogenic peptide B fragments and the LC-MS repeatability was validated using a commercial standard BSA-digest (Waters). For protein identification the peaks were transformed to MGF files with Progenesis LC-MS (version 2.4, Nonlinear Dynamics, Newcastle on Tyne, UK) and searched using Mascot (version 2.2, in-house server), Swissprot 2014_05 (545388 sequences, 193948795 residues) all taxonomy, and the following parameters: Enzyme: Trypsin, Peptide Mass Tolerance: 0.2 Da, Fragment Mass Tolerance: 0.5 Da, Max missed cleavages 1, fixed modification: Carbamidomethyl (C), variable modification: oxidation (M). The false discovery rate<5% was defined using the Decoy database search in Mascot. The protein score was calculated based on ion score cut-off 25 for individual peptides.

### Proteolytic cleavage of PSA-substrates

PSA (0.2–1 µM) and 0.5–1 µM of each protein substrate were incubated at 37°C for 22 h in TBS buffer. During the incubation, samples of 15 µl were taken at different time points: 0 min, 10 min, 30 min, 2 h, 5 h and 22 h, and stored at −20°C before further analysis. As a cleavage control, plasminogen was also incubated with 1 µM MMP-3 at 37°C for 22 h in TBS.

To confirm that the substrates were cleaved by PSA and not by possible contaminating proteases in the PSA preparation, we inhibited the proteolytic activity of PSA by one-hour preincubation at room temperature with 0.4 µM (two-fold molar excess) monoclonal antibody 5C7 that efficiently inhibits PSA, but not hK2 (a structurally similar protease and a common contaminant in PSA preparations) [38], [39]. After this preincubation protein substrates (1 µg for all, except for semenogelin I 0.6 µg) were added for 20 h incubation at 37°C.

To study the effect of the PSA-binding peptides on the proteolytic activity of PSA towards protein substrates, PSA (0.1 µg, i.e., final concentration 0.2 µM) was first preincubated for 30 min at room temperature with 1 or 5 µg of peptides B2 and C4 (46 or 230 µM and 43 or 213 µM, respectively). Then 0.3–2.5 µM protein substrates (i.e., 1 µg for all, except for semenogelin I 0.6 µg) were added to the PSA-peptide mixture (final volume 15 µl) and incubated at 37°C for 10 min (semenogelin I), 40 min (semenogelin II), 4 h (fibronectin and nidogen-1) or 20–22 h (galectin-3 and IGFBP-3). Appropriate incubation time for each substrate was chosen according to the degradation experiments without peptides (above). Each peptide (5 µg) was also included as a control without PSA.

Immediately after each incubation, reducing 4× SDS-loading buffer (8% SDS), containing 1 M β-mercaptoethanol or 75 mM dithiothreitol, was added to each sample and proteins were denatured at 70°C for 10 min. The samples were immediately analyzed by SDS-PAGE or frozen at −20°C. Frozen samples were heated again at 70°C before loading on the gel. The cleavage of protein substrates by PSA, i.e., the disappearance of the full-length protein bands, was quantified from scanned images of silver stained gels by the measurement of relative optical densities of the protein bands using ImageJ [40].

For N-terminal sequencing, 1 µM (10 µg) nidogen-1 was first incubated with 2 µM (5 µg) PSA for 22 h at 37°C. Then the resulting protein fragments were separated by SDS-PAGE and electroblotted onto polyvinylidene-difluoride (PVDF) membrane [41]. After staining with Coomassie Brilliant Blue the protein fragments were directly sequenced by Edman-degradation using the Procise 494A Sequencer (Perkin Elmer Applied Biosystems, Foster City, CA).

### SDS-PAGE, silver staining and Western blotting

Proteins were separated using the XCell SureLock Mini Cell system and pre-cast NuPAGE Novex 4–12% Bis-Tris Gels (both from Invitrogen, Carlsbad, CA). MES running buffer, pH 7.3, containing 50 mM MES [2-(N-morpholino)ethanesulfonic acid] (Sigma-Aldrich), 50 mM Tris base, 3.5 mM SDS and 1 mM EDTA was used. Dithiothreitol (0.5 mM) (Sigma-Aldrich) was added to the cathode buffer chamber.

Proteins were visualized either by silver staining or Western blotting. For silver staining (modified from [42]), the gel was fixed for one hour at room temperature in 30% ethanol, containing 0.5% acetic acid, and rinsed with 20% ethanol and water for 10 min each. Then the gel was sensitized with 0.02% sodium thiosulfate for 1 min and rinsed twice with water. After 30 min incubation in 0.2% AgNO_3_, the gel was rinsed briefly with water and proteins were detected with development solution, containing 700 µl/l of 37% formaldehyde, 0.001% sodium thiosulfate and 3% anhydrous Na_2_CO_3_. The development reaction was finished with stop solution containing 5% Tris and 2.5% acetic acid.

For Western blotting, proteins were transferred to an Immobilon-P membrane (Millipore) by the Trans Blot semi-dry transfer system (BioRad Laboratories, Hercules, CA). The membrane was blocked over night at 4°C with 1% bovine serum albumin (BSA) in TBS prior to incubation with 0.1 µg/ml goat anti-nidogen-1 polyclonal antibody (AF2570, R&D Systems), and detected with horseradish peroxidase-conjugated rabbit anti-goat IgG (P0449, Dako, Glostrup, Denmark) using enhanced chemiluminescence (ECL) detection reagents (GE Healthcare, Buckinghamshire, UK).

### Immunofluorometric assay for IGFBP-3

To study the degradation of IGFBP-3 by PSA, we used a sandwich-type immunofluorometric assay (IFMA) that measures only intact IGFBP-3, but not that cleaved by PSA [43], [44]. PSA (0.1 µg in 15 µl, i.e., 0.2 µM) and 1 µg (2.2 µM) IGFBP-3 were incubated together at 37°C for 24 h in TBS buffer, with and without 1 or 5 µg of peptides B2 and C4 (46 or 230 µM and 43 or 213 µM, respectively). After incubation, the samples were diluted 1∶1000 in IFMA assay buffer and IGFBP-3 concentrations were measured as previously described [43],[44].

### Cell culture and HUVEC tube formation assay

Human umbilical vein endothelial cells (HUVECs) were isolated from umbilical cord veins and grown in Endothelial Cell Growth Medium (Promocell, Heidelberg, Germany) with 2% Ultroser G BioSepra serum substitute (PALL BioSepra, Cergy, France) and growth factors EGF and bFGF. For tube formation, HUVECs were grown on top of Matrigel (BD Biosciences) as described previously [23], [29].

To study the effect of PSA-cleaved protein fragments, 0.08 µM nidogen-1 or 0.4 µM galectin-3 (10 µg/ml each) were first incubated with 1 µM PSA for 24 h at 37°C in PBS (Lonza). Then PSA activity was blocked by one-hour incubation at room temperature with 2 µM monoclonal antibody 5C7 [38]. The mixture containing nidogen-1 or galectin-3 fragments with PSA blocked by antibody or the same amount of intact nidogen-1 or galectin-3 were added together with the cells onto gelled Matrigel and the cells were allowed to form tubular networks for 20 h. Control wells contained either PBS or 1 µM PSA. After overnight incubation, cell culture medium was collected from PBS and PSA control wells for SDS-PAGE and Western blotting. Images of tubular networks (100×magnification) were taken using an Axiovert 200 inverted light microscope connected to an AxioCam digital camera and visualized by AxioVision software (all from Zeiss, Göttingen, Germany).

### Ethic statement

The isolation of HUVECs and the use of human seminal fluid for PSA purification were approved by the Institutional Review Board of Helsinki University Central Hospital. All participants gave a written informed consent for the use of the cells or seminal plasma for research.

## Results

### Characterization of PSA kinetics and effect of peptides on the enzymatic activity

**Figure 1:**
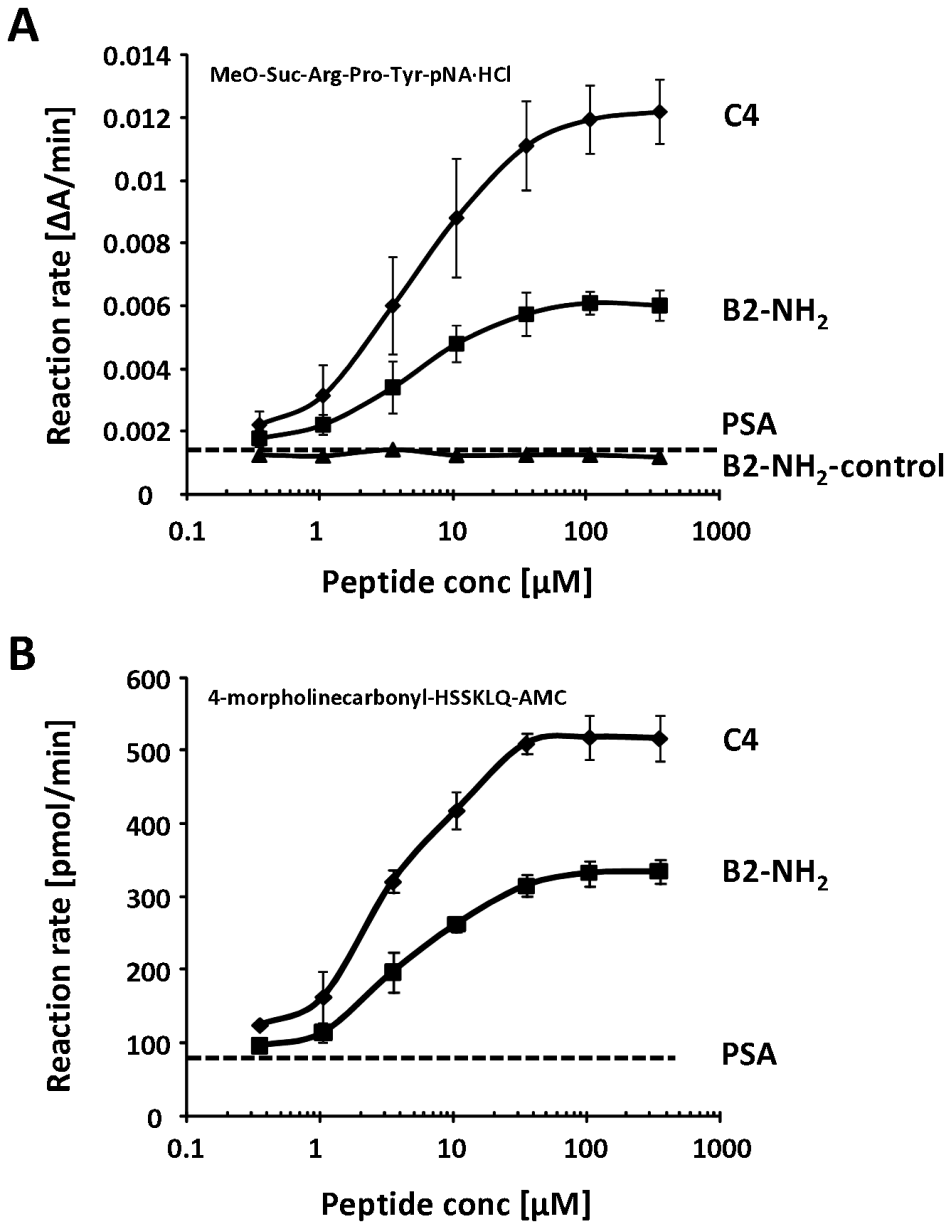
Peptides stimulate PSA activity towards small peptide substrates. Peptide C4 stimulated the activity of PSA (mean ± SD) more efficiently than peptide B2-NH_2_ towards (A) a chromogenic peptide substrate (n = 2) and (B) a fluorogenic peptide substrate (n = 3). Dashed lines indicate the activity of PSA without peptide stimulation.

The enzyme kinetics of PSA were determined with two different small peptide substrates (Table 1). These substrates were used to characterize the kinetic parameters also with peptides that stimulate the activity of PSA. Peptide C4 stimulated the activity of PSA towards chromogenic and fluorogenic peptide substrates six- to eight-fold, being more efficient than peptide B2-NH_2_, which stimulated PSA activity four-fold (Figure 1, Table 2). The effect of peptide B2 was similar to that of B2-NH_2_, while the inactive control peptide, B2-NH_2_-control, did not stimulate PSA activity at all (Figure 1A).

**Table 1:** Enzyme kinetics of PSA with small peptide substrates.

	K_m_	k_cat_	k_cat/_K_m_
	*(mM)*	*(s^−1^)*	*(s^−1^M^−1^)*
***Chromogenic substrate*** a	5.72±1.53	0.75±0.07	133.4±24.6
***Fluorogenic substrate*** b	1.58±0.04	0.19±0.09	122.0±59.4

The values represent mean ± SD of two experiments.

a S-2586, MeO-Suc-Arg-Pro-Tyr-pNA·HCl

b 4-morpholinecarbonyl-HSSKLQ-AMC

**Table 2:** Stimulation of PSA activity towards small peptide substrates with peptides B2-NH_2_ and C4.

	Stimulation	[peptide]_½Vmax_	[peptide]_2V_
	*(%)*	*(µM)*	*(µM)*
***Chromogenic substrate*** a			
**B2-NH_2_**	400±14	5.13±1.43	3.33±0.83
**C4**	785±7	5.62±2.17	1.55±0.54
***Fluorogenic substrate*** b			
**B2-NH_2_**	391±11	4.78±1.55	2.94±1.07
**C4**	610±8	3.72±0.50	1.36±0.25

Maximal stimulation capacity of the peptide as compared to PSA alone and peptide concentrations with half-maximal stimulation ([peptide]_½Vmax_) and two-fold stimulation of PSA activity ([peptide]_2V_) are shown. The values represent mean ± SD (n = 2 for chromogenic and n = 3 for fluorogenic substrate).

a,b As in Table 1.

### Binding affinity of the peptides to PSA

The binding kinetics and the affinity of the peptides (B2, B2-NH_2_, B2-NH_2_-control and C4) to PSA were studied by surface plasmon resonance. All peptides, except the inactive B2-NH_2_-control peptide, bound to PSA. However, the association or dissociation rate constants (k_a_ and k_d_), could not be estimated because the association of the peptides to and dissociation from PSA were too fast for reliable measurement.

The equilibrium dissociation constant (K_D_), i.e., the binding affinity, was calculated for each peptide from the equilibrium plot showing the binding (RU) as a function of peptide concentration (Figure S1). This showed that B2 (K_D_ = 73.6±20.3 µM, mean ± SD) and B2-NH_2_ (K_D_ = 56.5±5.3 µM) bound more strongly to PSA than peptide C4 (K_D_ = 197.8±30.5) (*p* = 0.034 and *p* = 0.021, respectively). There was no statistically significant difference in binding affinity between B2 and B2-NH_2_ (*p* = 0.29).

### Identification of nidogen-1 as a substrate for PSA

**Figure 2:**
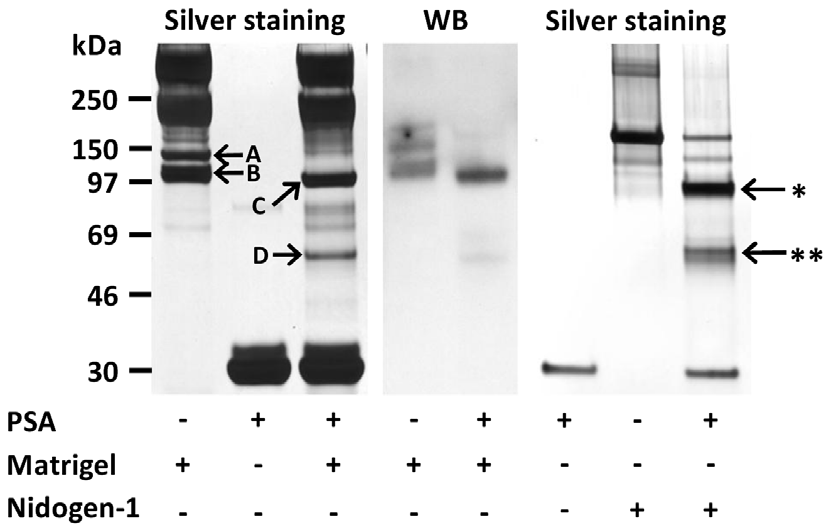
Degradation of nidogen-1 by PSA. PSA cleaved both mouse nidogen-1 in Matrigel and human recombinant nidogen-1. Mass spectrometry analysis identified nidogen-1 in the silver stained gel bands (arrows, left panel). Nidogen-1 bands of 140 kDa (A) and 110 kDa (B) disappeared and fragments of 90 kDa (C) and 55 kDa (D) appeared after 48 h incubation of diluted Matrigel with PSA at 37°C. Nidogen-1 cleavage by PSA in Matrigel was visualized by Western blotting with anti-nidogen-1 polyclonal antibody (middle panel). PSA (1 µM) cleaved recombinant human nidogen-1 (0.5 µM) into two fragments 85 kDa (arrow with *) and 55 kDa (arrow with **) during 20 h incubation at 37°C (right panel).

Matrigel, a basement membrane preparation of mouse origin, was incubated with PSA and the degradation products were analyzed by SDS-PAGE followed by silver staining. After incubation, 110 and 140 kDa bands disappeared, while new smaller bands of 55 and 90 kDa in size were observed (Figure 2). Mass spectrometric analysis of these bands identified nidogen-1 as a first hit with Mascot scores of 1057 (17 peptides from nidogen-1 identified), 1153 (18 peptides), 439 (9 peptides) and 1156 (17 peptides), respectively (Table S1). Furthermore, these bands reacted with nidogen-1 antibody in Western blotting (Figure 2). The cleavage of nidogen-1 by PSA was confirmed using recombinant nidogen-1 (Figure 2). Weak staining of full-length nidogen-1 was also observed by Western blotting in cell culture medium samples, collected after HUVEC tube formation assay performed on Matrigel, and nidogen-1 cleavage products were observed when PSA was present in the assay (data not shown).

The cleavage site(s) of PSA in nidogen-1 were determined by N-terminal sequencing of two fragments of recombinant nidogen-1 (55 and 85 kDa) resulting from incubation with PSA (Figure 2). The N-terminal amino acids were G/(S), Y, N, T, D for the 55 kDa fragment and G/(S), (Y), (N), (T), (D) for the 85 kDa fragment (amino acids in parenthesis were not identified with high reliability). Based on this, the cleavage site for PSA in nidogen-1 was determined to be GVVF^380^ ↓ SYNTD (numbering according to the UniProt sequence P14543).

### Cleavage of protein substrates and effect of stimulating peptides

**Figure 3:**
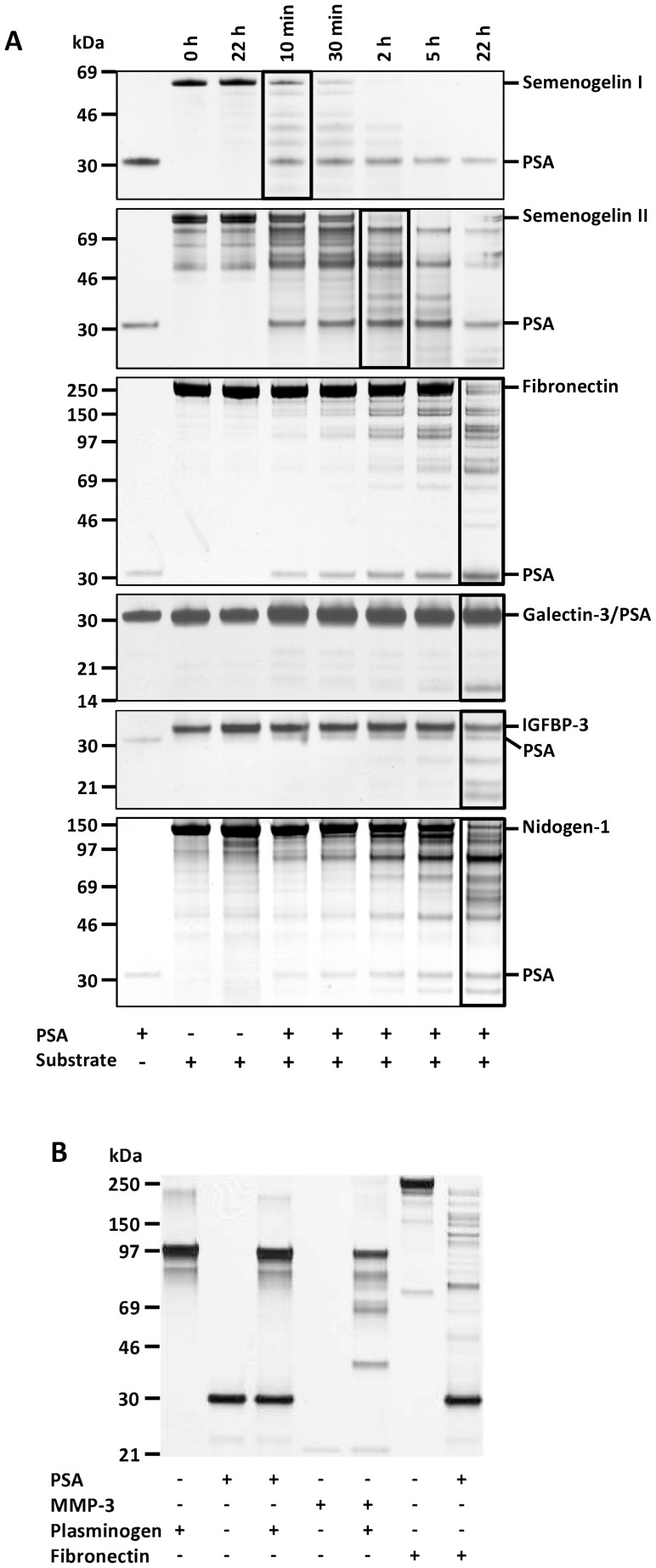
Cleavage of protein substrates by PSA. (A) Characterization of the proteolytic activity of PSA (0.2 µM) during 22 h incubation towards different protein substrates (1 µM each, except 0.5 µM semenogelin I) by SDS-PAGE and silver staining. Approximate molecular weights of the proteins are: PSA (28 kDa), semenogelin I (50 kDa), semenogelin II (63 kDa), fibronectin (220 kDa), galectin-3 (26 kDa), IGFBP-3 (30 kDa) and nidogen-1 (130 kDa). The lanes in which ∼50% of the proteins were cleaved are bordered. (B) 1 µM MMP-3 (22 kDa), but not PSA, cleaved 1 µM plasminogen (88 kDa). Also 0.5 M fibronectin was incubated with 1 µM PSA as a control (SDS-PAGE with silver staining).

Several protein substrates were incubated together with PSA and samples were drawn at different time points during incubation for up to 22 h. PSA cleaved its physiological substrates semenogelins very efficiently (Figure 3A). As determined by the disappearance of the full-length protein bands, half of the intact semenogelins I and II were degraded by PSA after 11 min and 80 min incubation, respectively. Fragments of fibronectin, galectin-3, IGFBP-3 and nidogen-1 could be detected after 2–5 h incubation with PSA, but extensive cleavage was readily seen only after overnight incubation. Thus, 50% of fibronectin was degraded in 17 h and of IGFBP-3 and nidogen-1 after 22 h.

Monoclonal antibody 5C7 inhibited the cleavage of all protein substrates (Figure S2 and data not shown). In contrast to MMP-3, PSA did not cleave plasminogen after 22 h incubation, even if different plasminogen preparations and PSA batches were used (Figure 3B).

**Figure 4:**
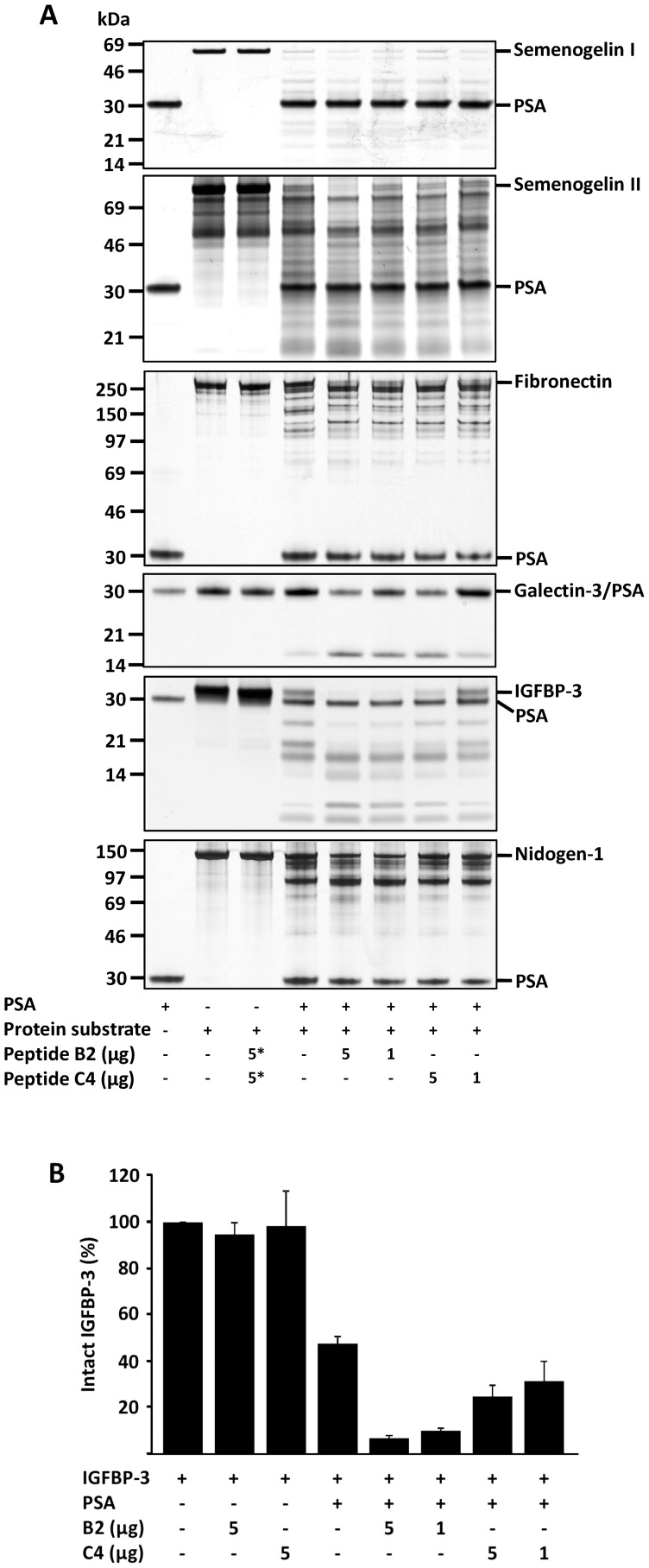
The effect of peptides on the proteolytic cleavage of different protein substrates by PSA. (A) Peptide B2 enhanced the activity of PSA towards all protein substrates more strongly than C4, except for fibronectin this could not be detected. The peptides were preincubated with 0.2 µM PSA for 30 min prior to addition of 0.3–2.5 µM protein substrates and the cleavage of the proteins was detected after 10 min (semenogelin I), 40 min (semenogelin II), 4 h (fibronectin and nidogen-1) and 20–22 h (galectin-3 and IGFBP-3) incubation at 37°C. The notation 5* indicates that 5 µg of either peptide (with semenogelins I and II peptide C4, and with the other proteins B2) was added to the control sample. (B) Concentration of intact IGFBP-3 after incubation with PSA and peptides shown in relation to IGFBP-3 control without added PSA as measured by immunofluorometric assay that recognizes only intact, not cleaved IGFBP-3 (n = 2, mean ± SD).

Peptides B2 and C4 stimulated the activity of PSA towards semenogelin I (10 min incubation with PSA), semenogelin II (40 min incubation), fibronectin and nidogen-1 (4 h incubation), and galectin-3 and IGFBP-3 (20–22 h incubation) (Figure 4A). The extent and the relative efficiency of the stimulation of PSA activity by the peptides were different from those observed with small peptide substrates, peptide B2 being more effective than C4 with protein substrates (except with fibronectin the cleavage was stimulated to a similar extent with B2 and C4). PSA decreased the content of intact IGFBP-3 by 50% in 22 h as measured by the immunofluorometric assay, which recognizes only full-length non-proteolyzed IGFBP-3, and both peptides stimulated the proteolytic activity of PSA towards IGFBP-3 further, peptide B2 being much more efficient than C4 (Figure 4B).

### Effect of nidogen-1 and galectin-3 on HUVEC tube formation

**Figure 5:**
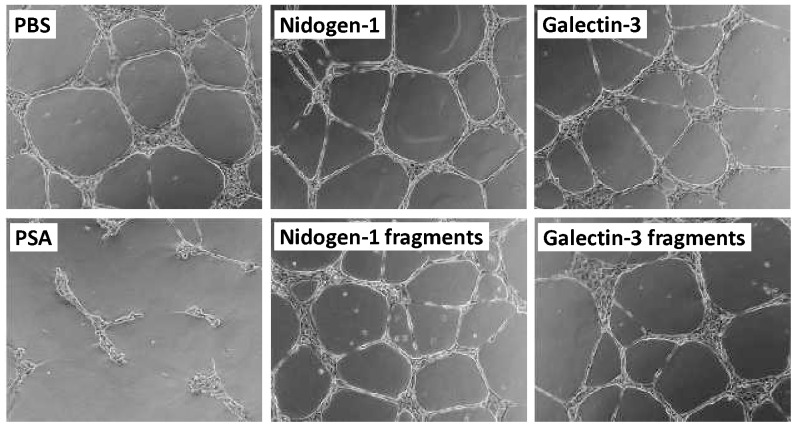
HUVEC tube formation assay with nidogen-1 and galectin-3. Nidogen-1 and galectin-3 or their PSA-generated fragments did not have any effect on tube formation. Control wells are with PBS and PSA. Images are representative examples from two separate experiments.

The effect of nidogen-1 and galectin-3 and their fragments generated by PSA was studied in the HUVEC tube formation assay, in which PSA reduced tube formation indicating decreased angiogenic potential of the cells (Figure 5). Neither PSA-produced fragments of nidogen-1 or galectin-3, nor their full-length forms, did have any effect on angiogenesis in this model.

## Discussion

The kallikrein-related peptidase (KLK) family comprises 15 members, most of which exert trypsin-like enzymatic activity [9], [14]. PSA has chymotrypsin-like activity, although its specificity is more restricted than that of chymotrypsin [45]. Furthermore, the proteolytic activity of PSA is much weaker than that of chymotrypsin or trypsin [14], [36]. KLK7 also exerts chymotrypsin-like activity, while KLK1, 10 and 11 have been reported to have both tryptic and chymotryptic activities [46].

Previously, several selective and efficient substrates for PSA have been described. We used a fluorogenic peptide substrate identified by Denmeade and coworkers (1997) [36]. The specificity constant k_cat/_K_m_, which is generally considered a measure of the efficiency of an enzyme, was about five-fold higher in our study compared to that reported by Denmeade *et al.* [36]. In previous studies, the k_cat/_K_m_ for PSA with the chromogenic substrate S-2586 has also varied, being somewhat lower than that we observed [15], [47]. These differences may be explained by higher activity of the highly pure active isoform of PSA used in this study [32], [33], differences in substrate preparations and/or minor differences in assay conditions.

The concentration of PSA in seminal fluid varies between ∼0.3 and 3 mg/ml (10–100 µM) and normally only trace amounts reach circulation [12]. Active PSA forms complexes with protease inhibitors and occurs in circulation mostly in an inactive form [10], [48]. In prostate cancer, the tissue architecture and the secretory pathway to the prostatic ducts are disrupted leading to increased leakage of PSA into extracellular fluid and circulation [10], [12], [49], but the expression of PSA is actually lower in cancer than in the normal prostate [50], [51]. In the extracellular fluid of prostate cancer tissue high PSA concentrations, around 2 µM, have been measured with about 90% of the PSA being enzymatically active [52]. The high concentrations of active PSA in prostatic tissue may have implications in cancer development and thus PSA could both enhance and inhibit the growth of prostate cancer at different stages of cancer development [13], [30].

While semenogelins I and II are considered to be the physiological substrates of PSA [16], [53], several other protein substrates have previously been reported to be degraded by PSA [9]. These proteins or their proteolytic fragments may either promote or inhibit cancer progression, e.g., the cleavage of fibronectin and laminin may be involved in cell invasion [25], [26], IGFBP-3 is associated with cell proliferation [44], [54], galectin-3 is involved in several processes including cell adhesion, proliferation, apoptosis and angiogenesis [55], [56] and plasminogen-derived angiostatin-like fragments inhibit angiogenesis [20]. In addition to these, PSA has been reported to cleave some unidentified protein components of the Matrigel basement membrane preparation [26], in which we now identified nidogen-1 as a new substrate for PSA. Nidogen-1 has a major structural role as a link between laminin and collagen type IV in the basement membrane [31], [57]. Several proteases including leukocyte elastase, mast cell chymase, plasma kallikrein, MMP-7, MMP-19 and cathepsin S have been reported to cleave mouse nidogen-1, while cathepsin S also cleaved recombinant human nidogen-1 [58]–[61]. As nidogen-1 is an essential linker molecule in basement membranes, its proteolytic cleavage may disrupt the basement membrane structure [58], [62].

In the present study, we compared the proteolytic activity of PSA towards several protein substrates. As shown previously, semenogelins are cleaved very effectively by PSA. Malm and coworkers (2000) reported that more than half of semenogelin I is degraded after 15 min incubation with PSA [16], [63] and we observed this after 11 min incubation. Proteolysis of the other protein substrates, i.e., fibronectin, IGFBP-3, galectin-3 and nidogen-1, took much longer, reflecting the high substrate specificity of PSA for semenogelins. Although degradation of proteins other than semenogelins is relatively slow, the concentration of PSA in the prostate is very high and, thus, the physiological relevance of the cleavage of other substrates by PSA cannot be ruled out. In this respect it is noteworthy, that the prostate also produces several other proteases, including trypsin and other KLKs, that are highly active [44], [64], [65]. Protease contamination in the PSA preparation used in this study was ruled out by using a monoclonal antibody that specifically inhibited the activity of PSA and prevented the proteolytic cleavage of all protein substrates studied.

PSA has been shown to cleave its substrates preferentially after amino acid residues tyrosine and glutamine and less commonly after leucine and other residues [45], [63], [66], [67]. Using N-terminal sequencing we identified one cleavage site of PSA in recombinant human nidogen-1, but most probably there are still one or more cleavage sites, as the same N-terminus was found in two fragments of different size. As other cleavages may take place, the resulting smaller fragments could be further degraded becoming undetectable with SDS-PAGE and silver staining. The identified cleavage site GVVF^380^ ↓ SYNTD is fairly typical for PSA, as there is phenylalanine (F) at the P1 position and serine (S) at the P1′ position [67], [68]. Interestingly, another protease with chymotrypsin-like specificity, mast-cell chymase, has been reported to cleave mouse nidogen-1 at the same site [58]. The cleavage site between phenylalanine and serine (amino acids 380 and 381 in the UniProt sequence P14543) is located between globular domains G1 and G2 in the link region of nidogen-1, which is highly sensitive to proteolysis [31], [58], [62]. The cleavage occurs just before domain G2 and thus PSA releases domain G1 and most of the link region from nidogen-1. Unlike domains G2 and G3, domain G1 is not involved in the interaction with laminin or collagen type IV, but it binds to fibulin-2, another crosslinking protein in the basement membrane [69]. However, fibulin-2 binds also to the G2 and G3 domains. Thus, the biological significance of the cleavage of nidogen-1 by PSA is unclear. On the contrary, the cleavage of nidogen-1 by cathepsin S occurred at three sites within the G2 and G3 domains. This was shown to abolish its binding to laminin and collagen type IV, and thus inhibited the linker function of nidogen-1 [61].

PSA has been shown to exert antiangiogenic activity both *in vitro* and *in vivo* [19], [21] and we have shown that enzymatic activity is needed for this function [23], [29]. However, the detailed mechanism of the antiangiogenic activity of PSA remains unclear. Some of the proteolytically produced fragments of extracellular matrix (ECM) proteins act as endogenous angiogenesis inhibitors [27] and may mediate the antiangiogenic effect of PSA. Previously, PSA has been reported to cleave plasminogen to angiostatin-like fragments that inhibit angiogenesis *in vitro* [20]. Although plasminogen was readily cleaved by MMP-3, different batches of active PSA purified in our laboratory were not found to generate any fragments using different plasminogen preparations. Moreover, when we treated HUVECs with PSA, the antiangiogenic effect was not associated with measurable levels of angiostatin in cell culture samples as measured by an ELISA immunoassay, which detects 100-fold lower levels of angiostatin than those reported to reduce tube formation and 10-fold lower levels needed for inhibition of migration [70], [71] (Mattsson et al. unpublished data). It is also noticeable that the reported PSA cleavage sites in plasminogen [20], i.e., after lysine (K) and glutamic acid (E), do not fit well with the known major cleavage sites of PSA according to the peptidase database MEROPS [67]. Thus it is possible that the PSA used in the original study was contaminated by other proteases that are able to cleave plasminogen. Indeed, several PSA preparations have been found to contain trypsin-like proteases [39].

Interestingly, galectin-3 has been reported to promote angiogenesis and its 21 kDa fragment was shown to inhibit angiogenesis induced by the full-length protein [55], [72]. Nidogen-1 may also play a role in angiogenesis as it is up-regulated in endothelial tip cells during blood vessel formation [73]. However, our results show that although PSA cleaves nidogen-1, overnight pre-incubation of gelled Matrigel with PSA, followed by inhibition of PSA with an antibody, did not inhibit endothelial cell tube formation (Mattsson et al. unpublished data). This shows that the antiangiogenic effect of PSA is most probably not due to a proteolytic cleavage of nidogen-1 in Matrigel. Moreover, nidogen-1 or galectin-3 or their fragments generated by PSA did not have any effect on angiogenesis in the tube formation assay. It is noteworthy, that this assay was optimized for angiogenesis inhibition and therefore we did not detect any potential stimulation of angiogenesis by full-length galectin-3.

The synthetic peptides that we originally identified by phage display, stimulate the proteolytic activity of PSA most likely by binding near its active site [28], [74]. It has been predicted that binding of the peptides stabilizes the kallikrein loop of PSA in an open conformation and this would increase the proteolytic activity [75]. When measured with chromogenic and fluorometric assay, peptides B2, B2-NH_2_ and C4 greatly enhanced PSA activity towards two different peptide substrates. Peptides B2 and B2-NH_2_ were equipotent, while peptide C4 was more active in this respect, which is in keeping with earlier observations with the chromogenic substrate [28], [32]. These peptides have also been found to enhance the antiangiogenic activity of PSA in the endothelial cell tube formation assay, when added together with PSA in the cell culture medium [29].

We found that the PSA-stimulating peptides B2 and C4 enhanced the activity of PSA towards all protein substrates studied, but their relative efficiency was different from that found with small peptide substrates. With all studied protein substrates, except for fibronectin, peptide B2 was more efficient in stimulating PSA activity, while C4 was more efficient with peptide substrates. This may be explained by differences in the size and structure of the PSA substrates. Although with some of the protein substrates the peptides stimulated the activity of PSA dose-dependently, we could not observe such dose-dependent stimulation when nidogen-1 or fibronectin were used as substrates. The reason for this is unclear, but may be due to high peptide concentrations used.

The interaction of the peptides with PSA was found to be very fast as studied by surface plasmon resonance. Previously, when these peptides were produced as fusion proteins with glutathione-S-transferase (GST), their equilibrium dissociation constants (K_D_) with PSA were about 20- to 60-fold lower than in our study, i.e., GST-peptides bound more strongly to PSA [28]. This is explained by multivalent binding, as part of the GST-peptides existed as multimers. In the present study, peptides B2 and B2-NH_2_ bound more strongly to PSA than C4, but with GST-peptides the opposite was observed [28].

## Conclusions

In addition to efficient proteolysis of semenogelins, PSA exerted weak proteolytic activity towards the other protein substrates studied. A basement membrane protein, nidogen-1, was identified as a new substrate for PSA. The cleavage of nidogen-1 by PSA may lead to disruption of basement membrane structure and may facilitate cancer cell invasion, but our results suggest that PSA-generated nidogen-1 or galectin-3 fragments do not mediate the antiangiogenic function of PSA. Thus, the mechanism of the antiangiogenic properties of PSA remains to be clarified. Peptides that stimulate the activity of PSA towards small peptide substrates also enhanced the cleavage of protein substrates by PSA. Interestingly, the PSA-activity stimulating peptide that showed less efficient stimulation with peptide substrates was more efficient with protein substrates.

## Supporting Information

Figure S1 Interaction of the peptides with PSA as studied by surface plasmon resonance. The representative examples of sensorgrams and the corresponding equilibrium plots showing the peptide binding (resonance unit, RU) as a function of the concentration of (A) B2-NH_2_, (B) B2, (C) C4, and (D) B2-NH_2_-control. The dissociation constants (K_D_) were derived from the equilibrium plots from three to four separate experiments each with two replicates.
(PDF)

Figure S2 The cleavage of protein substrates is inhibited by a monoclonal antibody specific for PSA. PSA (0.2 µM) was preincubated for one hour with mAb 5C7 (0.4 µM) prior to incubation for 20 h with protein substrates (A) semenogelins I and II, or (B) fibronectin and nidogen-1.
(PDF)

Table S1 The nidogen-1 peptide sequences identified by Mascot search in different gel bands (shown in Figure 2). All peptides with ions score >25 are shown.
(PDF)
