# Supplementary material for: Proteolytic Activity of Prostate-Specific Antigen (PSA) towards Protein Substrates and Effect of Peptides Stimulating PSA Activity
Source: PLoS One. 2014 Sep 19;9(9):e107819. doi: 10.1371/journal.pone.0107819 (PMC4169579; doi:10.1371/journal.pone.0107819)

Figure S1. Interaction of the peptides with PSA as studied by surface plasmon resonance. The representative examples of sensorgrams and the corresponding equilibrium plots showing the peptide binding (resonance unit, RU) as a function of the concentration of (A) B2-NH_2, (B) B2, (C) C4, and (D) B2-NH_2-control. The dissociation constants (K_D) were derived from the equilibrium plots from three to four separate experiments each with two replicates.

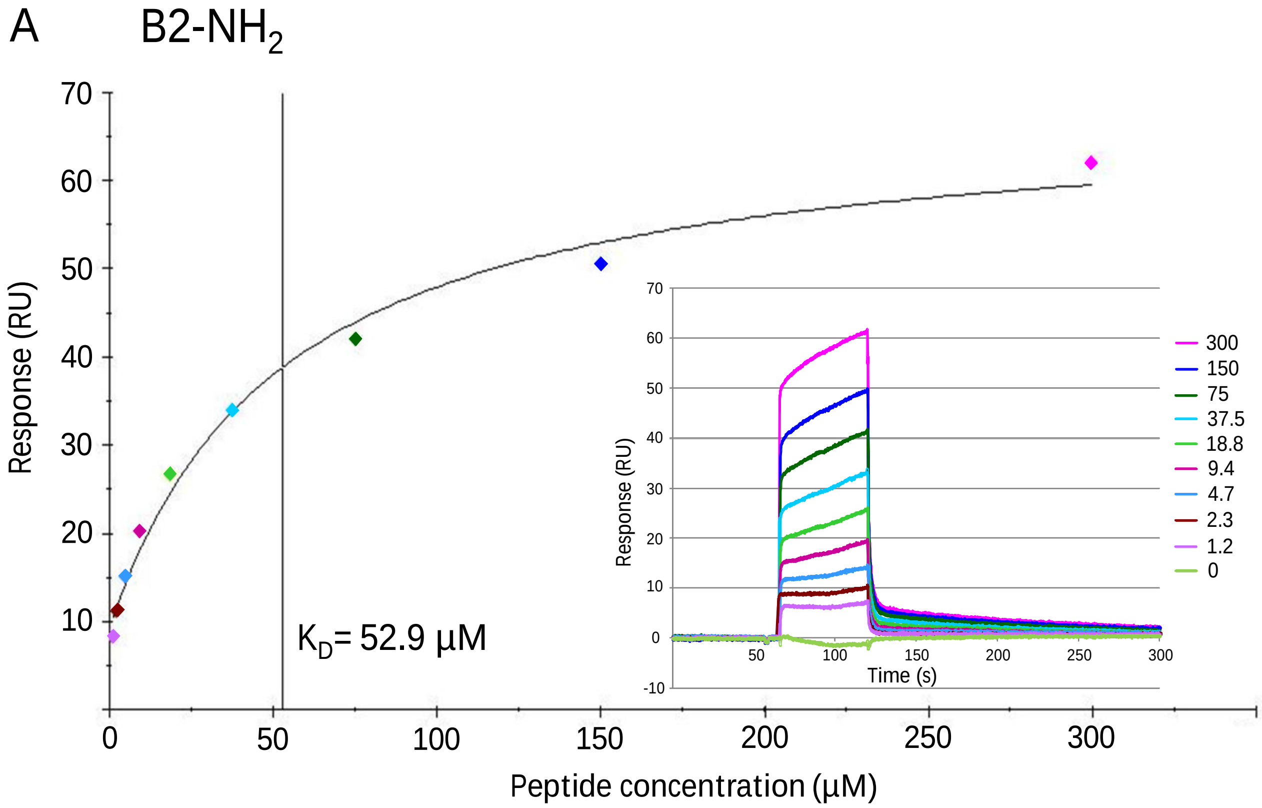

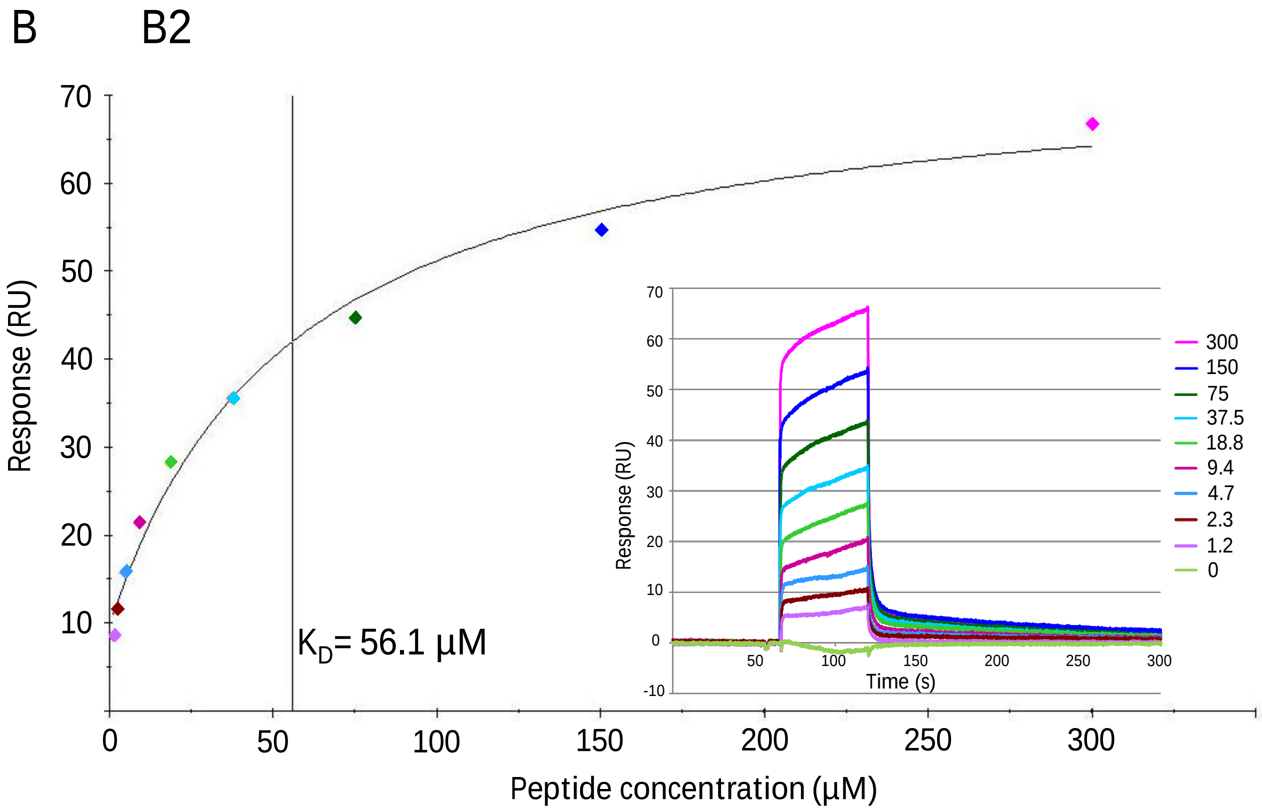

C C4

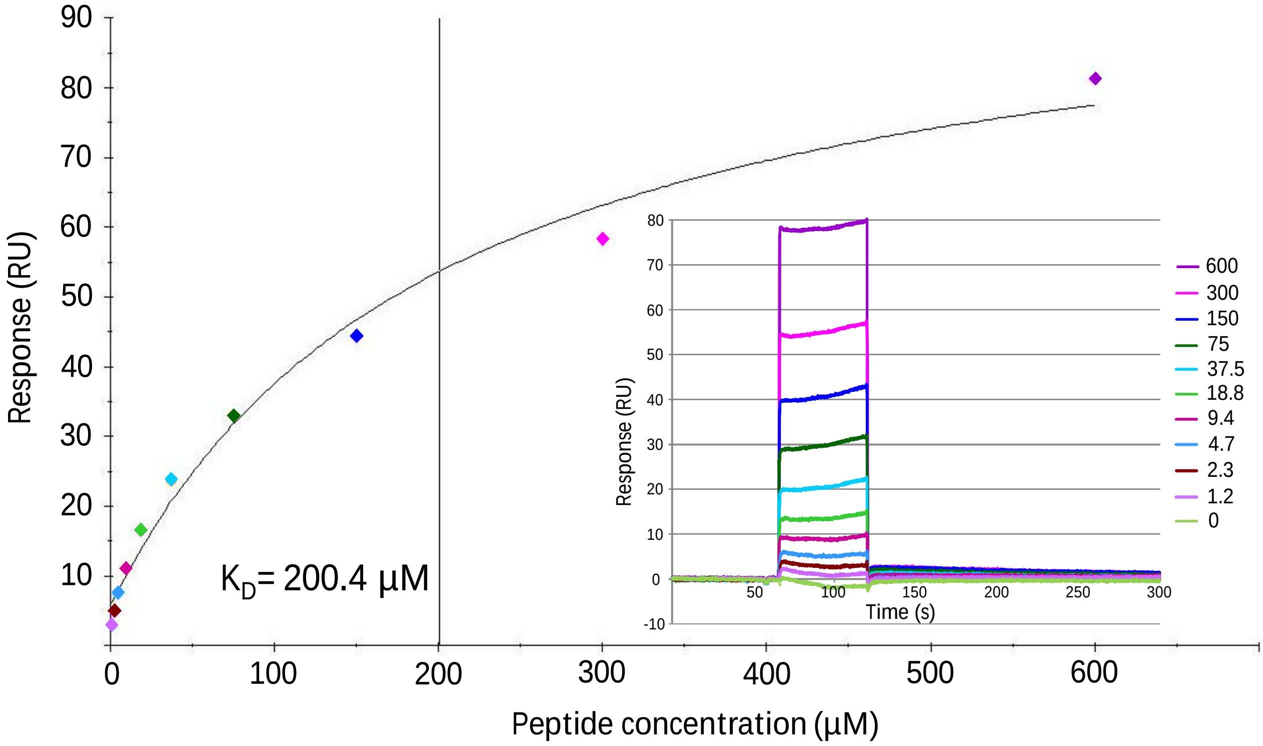

D B2-NH_2-control

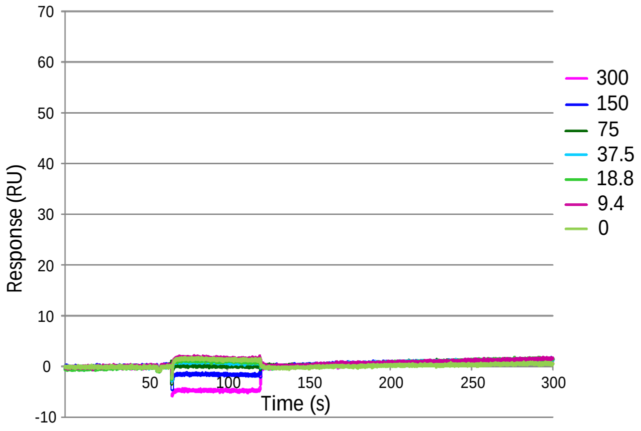

Supplement: Figure S1 — Interaction of the peptides with PSA as studied by surface plasmon resonance. The representative examples of sensorgrams and the corresponding equilibrium plots showing the peptide binding (resonance unit, RU) as a function of the concentration of (A) B2-NH2, (B) B2, (C) C4, and (D) B2-NH2-control. The dissociation constants (KD) were derived from the equilibrium plots from three to four separate experiments each with two replicates. (PDF) [file pone.0107819.s001.pdf]
