# Supplementary material for: Proteolytic Activity of Prostate-Specific Antigen (PSA) towards Protein Substrates and Effect of Peptides Stimulating PSA Activity
Source: PLoS One. 2014 Sep 19;9(9):e107819. doi: 10.1371/journal.pone.0107819 (PMC4169579; doi:10.1371/journal.pone.0107819)
Supplement: Figure S2 — The cleavage of protein substrates is inhibited by a monoclonal antibody specific for PSA. PSA (0.2 µM) was preincubated for one hour with mAb 5C7 (0.4 µM) prior to incubation for 20 h with protein substrates (A) semenogelins I and II, or (B) fibronectin and nidogen-1. (PDF) [file pone.0107819.s002.pdf]

Figure S2. The cleavage of protein substrates is inhibited by a monoclonal antibody specific for PSA. PSA (0.2 µM) was preincubated for one hour with mAb 5C7 (0.4 µM) prior to incubation for 20 h with protein substrates (A) semenogelins I and II, or (B) fibronectin and nidogen-1.

A

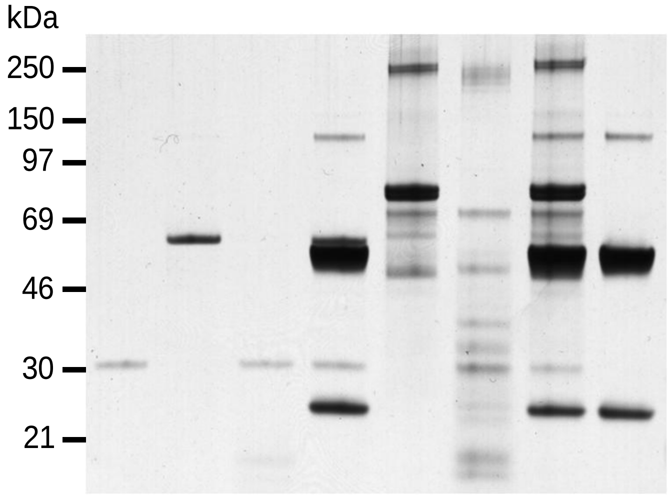

PSA	+	-	+	+	-	+	+	-
Semenogelin I	-	+	+	+	-	-	-	-
Semenogelin II	-	-	-	-	+	+	+	-
mAb 5C7	-	-	-	+	-	-	+	+

B

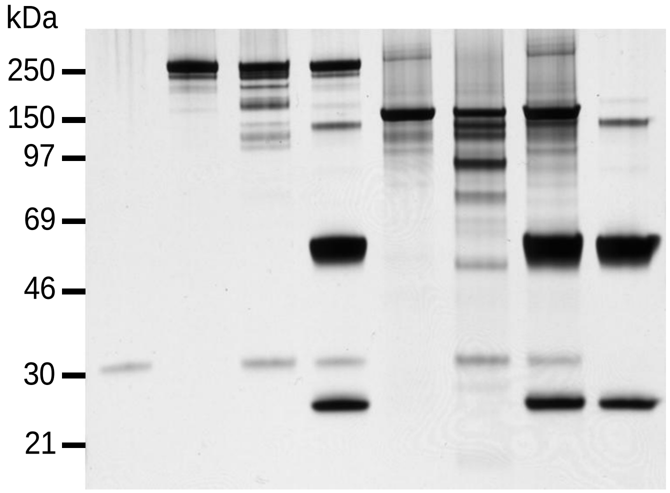

PSA	+	-	+	+	-	+	+	-
Fibronectin	-	+	+	+	-	-	-	-
Nidogen-1	-	-	-	-	+	+	+	-
mAb 5C7	-	-	-	+	-	-	+	+
