# Supplementary material for: Proteolytic Activity of Prostate-Specific Antigen (PSA) towards Protein Substrates and Effect of Peptides Stimulating PSA Activity
Source: PLoS One. 2014 Sep 19;9(9):e107819. doi: 10.1371/journal.pone.0107819 (PMC4169579; doi:10.1371/journal.pone.0107819)
Supplement: Table S1 — The nidogen-1 peptide sequences identified by Mascot search in different gel bands (shown in Figure 2). All peptides with ions score >25 are shown. (PDF) [file pone.0107819.s003.pdf]

Table S1.

The nidogen-1 peptide sequences identified by Mascot search in different gel bands (shown in Figure 2). All peptides with ions score > 25 are shown.

A) 140 kDa band

Sequence	Peptide Ions Score
GDGQTCYDIDECSEQPSR	130
CPDNTLGVDCIER	94
VLEGLQYPFAVTSYGK	88
VLFDTGLVNPR	83
TIFWTDSQLDR	81
QDLGSPEGIALDHLGR	76
TNSVIAMDLAISK	75
VVYWTDISEPSIGR	73
CECVEGYHFSDR	57
AECLNPAQPGR	56
RVLFDTGLVNPR	55
QCVAEGSPQR	51
SSNAGHQGVWVFEIGSPATAK	48
GYPDPHNVPR	47
VIIGLAFDCVDK	42
ASLHGGEPTTIIR	36
GTCVAAEDQRPINYCETGLHNCDIPQR	35
RGYPDPHNVPR	25

B) 110 kDa band

Sequence	Peptide Ions Score
VLEGLQYPFAVTSYGK	91
TNSVIAMDLAISK	88
GDGQTCYDIDECSEQPSR	82
TIFWTDSQLDR	81
VLFDTGLVNPR	80
YALSNSIGPVR	79
GNLYWTDWNR	70
CECVEGYHFSDR	69
EYTVMEPDQDGAAPSHTHIYQWR	68
NGFSITGGEFTR	60
AECLNPAQPGR	60
QAEVTFLGHPGK	54
QCVAEGSPQR	50
ASLHGGEPTTIIR	43
CPDNTLGVDCIER	28

VIIGLAFDCVDK	28
GIVTDPVR	27

C) 90 kDa band

**Sequence**	**Peptide Ions Score**
GDGQTCYDIDECSEQPSR	130
VLEGLQYPFAVTSYGK	84
VLFDTGLVNPR	80
YALSNSIGPVR	79
TNSVIAMDLAISK	78
VIIGLAFDCVDK	77
QDLGSPEGIALDHLGR	72
ASLHGGEPTTIIR	70
GNLYWTDWNR	69
IETSHMDGTNR	68
VVYWTDISEPSIGR	65
AECLNPAQPGR	63
CECVEGYHFSDR	60
TIFWTDSQLDR	52
QCVAEGSPQR	47
IETSHMDGTNRR	33
EYTVMEPDQDGAAPSHTHIYQWR	29

D) 55 kDa band

**Sequence**	**Peptide Ions Score**
VLEGLQYPFAVTSYGK	71
YALSNSIGPVR	71
VVYWTDISEPSIGR	65
CPDNTLGVDCIER	42
AECLNPAQPGR	40
TNSVIAMDLAISK	40
QCVAEGSPQR	39
KDESQVPAVVGFSK	37
QDLGSPEGIALDHLGR	32
